# Efficacy and safety of Guanylyl cyclase C agonists (linaclotide and plecanatide) in patients with irritable bowel syndrome with constipation: a systematic review and meta-analysis of randomized controlled trials

**DOI:** 10.3389/fphar.2026.1761301

**Published:** 2026-04-10

**Authors:** Zihao Zhou, Yuanlin Li, Yuyuan Tu, Meiling He, Defu Liao, Ziyan He, Zhiren Liu, Boyu Li, Zugang Zhou, Shuangchun Ai

**Affiliations:** 1 School of Health and Rehabilitation, Chengdu University of Traditional Chinese Medicine, Chengdu, China; 2 Department of Rehabilitation, Mianyang Hospital of Traditional Chinese Medicine, Mianyang, China

**Keywords:** constipation, irritable bowel syndrome, guanylyl cyclase C agonists, linaclotide, plecanatide, randomized controlled trials, meta-analysis

## Abstract

**Background and Objectives:**

Guanylyl cyclase C (GCC) agonists, including linaclotide and plecanatide, induce prosecretory and analgesic effects by elevating cGMP levels in the intestinal lumen, rendering them significant treatment alternatives for constipation-predominant irritable bowel syndrome (IBS-C). Nonetheless, disparities in efficacy and safety across medications and dosages necessitate a thorough assessment using systematic reviews and meta-analyses, primarily focusing on composite efficacy endpoints approved by the U.S. Food and Drug Administration (FDA).

**Methods:**

A systematic search of PubMed, Embase, the Cochrane Library, Web of Science, and ClinicalTrials.gov databases was conducted to identify randomized controlled trials comparing the aforementioned GCC agonists with placebo for the treatment of IBS-C. The search timeframe spans from the establishment of each database to 7 September 2025. Three researchers independently performed literature screening, data extraction, and methodological quality assessment (using the Cochrane Risk of Bias Assessment Tool). The primary endpoint is the proportion of patients achieving the FDA composite endpoint. Secondary endpoints include indicators related to abdominal pain and constipation. The safety outcome was the incidence of diarrhea. Statistical analyses were performed using RevMan 5.4 and Stata 18.0 software.

**Results:**

A total of 6 linaclotide and 3 plecanatide trials were included, involving 5,718 patients with IBS-C. Compared with placebo, GCC agonists significantly increased the proportion of patients achieving the FDA composite endpoint (linaclotide 290 μg [relative risk (RR) = 1.78, 95% CI 1.51–2.09]; plecanatide 3 mg [RR = 1.63, 95% CI 1.35–1.96]; plecanatide 6 mg [RR = 1.67, 95% CI 1.36–2.05]). GCC agonists also demonstrated significant advantages across all secondary outcome measures. However, the incidence of diarrhea was significantly higher in both drug groups compared to the placebo group (linaclotide 290 μg [relative risk (RR) = 6.20, 95% CI 4.39–8.76]; plecanatide 3 mg [RR = 5.29, 95% CI 1.59–17.64]; plecanatide 6 mg [RR = 4.00, 95% CI 1.52–10.51]).

**Conclusion:**

Linaclotide and plecanatide are effective medications for treating IBS-C, significantly improving both abdominal pain and constipation symptoms while increasing the risk of diarrhea. Their efficacy and safety should be carefully weighed when used clinically.

**Systematic Review Registration:**

https://www.crd.york.ac.uk/PROSPERO/view/CRD420251168079, Identifier CRD420251168079.

## 1 Introduction

Irritable bowel syndrome (IBS) is a functional gastrointestinal illness marked by enduring chronic abdominal pain or discomfort and alterations in bowel habits (Drossman, 2016). The global prevalence of this condition is estimated to be between 9% and 16%, with a significantly higher incidence in women, whose risk is nearly double that of men (Lovell and Ford, 2012). Approximately one-third of individuals with IBS are classified as having constipation-predominant irritable bowel syndrome (IBS-C) (Lovell and Ford, 2012; Longstreth et al., 2006; Drossman et al., 2002; Hungin et al., 2003).

IBS-C is characterized by abdominal symptoms such as pain and bloating, reduced stool frequency, hard or lumpy stools, straining during bowel movements, and a feeling of incomplete evacuation. Among these, difficulty passing stool and abdominal pain are the predominant symptoms, with prevalence rates reaching 75% and 66%, respectively, in the BURDEN IBS-C study (Quigley et al., 2018). The Rome IV criteria require recurrent abdominal pain at least once per week, accompanied by at least two of the following: altered stool consistency, altered stool frequency, or symptoms related to bowel movements (Lacy et al., 2016). The pathophysiological mechanisms of IBS-C are complex and multifactorial, involving dysfunction of the brain-gut axis, visceral hypersensitivity, low-grade intestinal inflammation, and gut microbiota dysbiosis (Pezzone et al., 2005; Chan et al., 2001; Sinhamahapatra et al., 2001; Naliboff et al., 2001; Kwan et al., 2005; Brierley and Linden, 2014; Grundy et al., 2019; Lacy et al., 2021; Mayer et al., 2015). These symptoms significantly impair patients’ quality of life, restricting daily activities and reducing work efficiency (Drossman et al., 2009; Ballou et al., 2019). Current clinical management of IBS-C includes lifestyle modifications (increased physical activity (Jin, 2014; Ford et al., 2018; Serra et al., 2020), low FODMAP diet (Domingo and Sánchez, 2020), fiber and probiotic supplementation (Isakov et al., 2013; Mezzasalma et al., 2016), and pharmacological therapy with osmotic laxatives, stimulant laxatives, and stool softeners (Sayuk et al., 2022). However, traditional treatments have considerable limitations: lifestyle interventions show inconsistent results with poor compliance, and laxatives often fail to adequately address abdominal symptoms (Ford et al., 2018). Consequently, many patients report ongoing symptoms and low satisfaction with existing therapies (Drossman et al., 2009; Harris et al., 2017), highlighting a significant unmet clinical need.

Guanylyl cyclase C (GCC) agonists represent a novel targeted therapy addressing IBS-C pathophysiology, with linaclotide and plecanatide as representative agents. They stimulate GCC receptors on intestinal epithelial cells, increasing intracellular and extracellular cyclic guanosine monophosphate (cGMP) levels. This activates protein kinase II, opening the cystic fibrosis transmembrane conductance regulator (CFTR) chloride channel, enhancing chloride and bicarbonate secretion into the intestinal lumen. Water then permeates the lumen by osmosis, hydrating stool (Vaandrager et al., 1998; Toriano et al., 2011; Busby et al., 2010), expediting colonic transit, and facilitating evacuation (Bryant et al., 2010; Eutamene et al., 2010). Additionally, extracellular cGMP directly influences intestinal nociceptive nerve terminals, reducing visceral hypersensitivity and abdominal pain (Cas et al., 2013; Grundy et al., 2018). Linaclotide, a synthetic 14-amino acid peptide, was approved by the FDA in 2012 for adult IBS-C at a dosage of 290 μg once daily. Plecanatide, a synthetic 16-amino acid peptide analog of human uroguanylin, received FDA approval in 2017 for adult IBS-C at 3 mg once daily, although the 6 mg dosage was also thoroughly evaluated during clinical development.

Despite numerous high-quality randomized controlled trials (RCTs) confirming the efficacy and safety of linaclotide and plecanatide for IBS-C (Brenner et al., 2023; Brenner et al., 2018), several evidence gaps remain. Direct comparative data between these two drugs—with similar mechanisms but distinct molecular structures and pharmacokinetics—are insufficient. Furthermore, the use of diverse efficacy endpoints across RCTs complicates direct comparisons. Therefore, a comprehensive evaluation using the current “gold standard”—the FDA composite efficacy endpoint (patients meeting both criteria for at least 6 weeks within the same week of the 12-week treatment period: (Drossman, 2016) mean daily abdominal pain score improvement ≥30% from baseline, (Lovell and Ford, 2012) increase in CSBM ≥1 from baseline)—is necessary. This systematic review and meta-analysis aimed to synthesize the highest-quality randomized controlled trial evidence, utilizing uniform and rigorous standards to deliver precise, comprehensive, and actionable evidence-based guidance for the clinical use of GCC agonists in treating IBS-C.

## 2 Methods

This study was registered with PROSPERO, registration number CRD420251168079. It was conducted according to the Preferred Reporting Items for Systematic Evaluation and Meta-Analyses (PRISMA) (Supplementary Appendix Table 1) (Page et al., 2021).

### 2.1 Search strategy

From the time of library construction to 7 September 2025, three review authors independently searched PubMed, Embase, the Cochrane Library, Web of Science, and ClinicalTrials.gov. The search terms included irritable bowel syndrome, constipation, Guanylyl cyclase-C agonist, linaclotide, plecanatide, and randomized controlled trial. The search terms are documented in detail in the Supplementary Appendix Table 2. In addition, we manually checked all reference lists of the retrieved papers and asked experts for any potentially relevant studies.

### 2.2 Inclusion and exclusion criteria (using the PICOS framework)

1. Participants (P): Adult patients (≥18 years) with IBS-C meeting Rome II or Rome III diagnostic criteria.
2. Interventions (I): Linaclotide 290 μg once daily, or plecanatide 3 mg once daily, or plecanatide 6 mg once daily, for a duration ≥12 weeks.
3. Comparisons (C): Placebo treatment.
4. Outcomes (O): Reported at least one of the primary or secondary outcomes specified in this paper.
5. Study design (S): Double-blind randomized controlled trials.

Exclusion criteria: (a) Conference reports, abstracts, animal studies, and replication studies; (b) Manuscripts lacking objective data or where data cannot be extracted, and where full-text retrieval remains impossible after contacting the corresponding author; (c) Studies related to children, organic constipation, or other diseases; (d) Non-English literature.

### 2.3 Study selection

Two researchers (Z Z and Y L) independently conducted literature screening and data extraction using EndNote X9. After removing duplicates, animal studies, and review articles, non-compliant literature was excluded via title and abstract screening, and the predefined inclusion and exclusion criteria were used to finalize the selection. Full-text articles requiring further evaluation were assessed and selected for inclusion in the analysis. Discrepancies were resolved by consulting and discussing with a third researcher (Y T).

### 2.4 Data extraction

Data extraction is performed in Excel using pre-designed standardized data extraction tables. Extracted independently by three researchers and cross-checked. Extracted information included: (a) Basic information: first author, publication year, sample size, study duration, intervention drug, and dose; (b) Participant baseline characteristics; (c) Data related to outcome measures (dichotomous or continuous variables); (d) Risk of bias assessment: randomization method, allocation concealment, and other relevant criteria.

### 2.5 Risk of bias assessment

Two blinded independent researchers employed the Cochrane Risk of Bias Tool (ROB) 2.0 (Jørgensen et al., 2016), to scrutinize the quality of included studies. The ROB 2.0 program evaluates five essential domains of potential bias: randomization, variations from intended interventions, missing outcome data, outcome measurement, and selective result reporting. When discrepancies occurred, a third researcher was involved to facilitate consensus.

### 2.6 Certainty of the evidence

The rating of recommendations is grounded in the GRADE (Grading of Recommendations Assessment, Development, and Evaluation) methodology, in which the certainty of the evidence is categorized as “very low,” “low,” “medium,” or “high” (Balshem et al., 2011). The quality of randomized controlled trials is high, whereas that of observational studies is low. Research quality can be lowered by five factors: limitations, inconsistency, indirectness, imprecision, and publication bias (Guyatt et al., 2011).

### 2.7 Data analysis

The statistical significance threshold was set at p < 0.05, and the data were integrated with RevMan 5.4 and Stata 18.0. The outcomes of this study were binary variables and continuous variables, and for each effect size, the researchers calculated 95% confidence intervals (CIs). Overall relative risks (RR) were calculated for dichotomous data. For continuous data included in the studies, which were assessed using the same scale and units, the results were synthesized using the weighted mean difference (WMD). Because this study aggregated data across different drugs (linaclotide and plecanatide), doses, and countries, heterogeneity in the underlying treatment effects was inevitable. A random-effects model was therefore adopted for all primary analyses. Forest plots were used to display the combined estimates. Sensitivity analyses were used to explore the causes of heterogeneity. Meta-regression analysis was used to test for differences between subgroups (Begg and Mazumdar, 1994; Stuck et al., 1998).

## 3 Results

### 3.1 Selection and inclusion of studies

**FIGURE 1:**
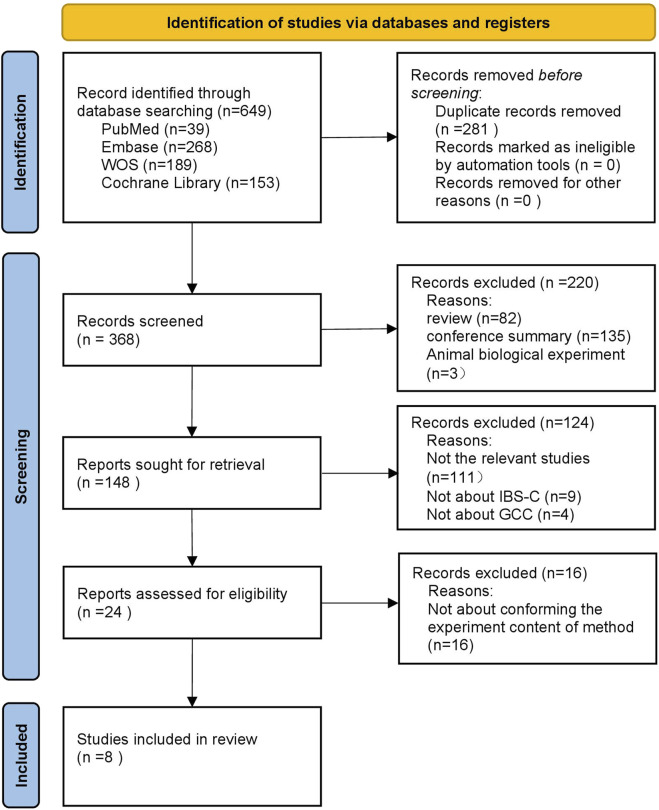
Project for Reporting of Systematic Evaluations and Meta-Analyses (PRISMA) flowchart.

An initial search of 649 studies was conducted through the above databases. Through multiple screening steps, eight studies were identified. Notably, the study by Brenner et al. encompassed two separate clinical trials, resulting in a total of nine clinical trials being included in the meta-analysis. (Brenner et al., 2018; Rao et al., 2012; Chey et al., 2012; Yang et al., 2018; Chang et al., 2021; Johnston et al., 2010; Chey et al., 2021; Miner et al., 2014). Examples include exclusion of duplicates, selection of literature types, systematic review of title and abstract competitions, and review of full text (Figure 1).

### 3.2 Characteristics of the included trials

A total of nine clinical trials were included in this study (Brenner et al., 2018; Rao et al., 2012; Chey et al., 2012; Yang et al., 2018; Chang et al., 2021; Johnston et al., 2010; Chey et al., 2021; Miner et al., 2014). The study was conducted primarily in the United States, Canada, China, Australia, and New Zealand. Participants met Rome II or Rome III criteria, totaling 5,718 individuals (as no studies employing the Rome IV criteria were identified during the database search, the inclusion criteria were restricted to studies that utilized the Rome II or Rome III criteria.). The experimental group comprised 3,221 participants, while the control group included 2,497 participants. The population was predominantly female (71.8%–91.7%), with an average age ranging from 41 to 47 years. The treatment cycle was primarily 12 weeks, with one case extended to 26 weeks. Patients’ baseline abdominal pain scores (measured on a 0–10 numerical rating scale) were generally moderate to severe, with mean scores ranging from 5.2 to 6.6 across the study groups. The patients’ baseline complete spontaneous bowel movement (CSBM) frequency was extremely low, with mean values across all study groups ranging from 0.2 to 0.34 times per week, clearly reflecting the core features of abdominal pain and defecation difficulty in IBS-C patients.

Of the nine trials, six used linaclotide (Rao et al., 2012; Chey et al., 2012; Yang et al., 2018; Chang et al., 2021; Johnston et al., 2010; Chey et al., 2021), and three used plecanatide (Brenner et al., 2018; Miner et al., 2014). In trials using plecanatide, 2 included doses of 3 mg and 6 mg (Brenner et al., 2018), while 1 included only 3 mg (Miner et al., 2014). Eight trials reported the FDA composite endpoint, a key indicator (Brenner et al., 2018; Rao et al., 2012; Chey et al., 2012; Yang et al., 2018; Chang et al., 2021; Chey et al., 2021; Miner et al., 2014). Six trials reported the proportion of patients who were abdominal pain responders and CSBM responders (Brenner et al., 2018; Rao et al., 2012; Chey et al., 2012; Chang et al., 2021; Chey et al., 2021). Five trials examined bowel movements within 24 h after the first dose (Brenner et al., 2018; Yang et al., 2018; Chang et al., 2021; Johnston et al., 2010). Eight trials evaluated changes in abdominal pain scores from baseline (Brenner et al., 2018; Rao et al., 2012; Chey et al., 2012; Yang et al., 2018; Chang et al., 2021; Chey et al., 2021; Miner et al., 2014). All trials reported two outcome measures: the change in CSBM frequency from baseline and the incidence of diarrhea (Brenner et al., 2018; Rao et al., 2012; Chey et al., 2012; Yang et al., 2018; Chang et al., 2021; Johnston et al., 2010; Chey et al., 2021; Miner et al., 2014). The specific basic characteristics of the included trials are shown in Table 1.

**TABLE 1:** Characteristics of included trials.

Study (year)	Country; sites	Diagnostic criteria used for IBS	Participants (Female%)	Age (years), mean (range/SD)	Abdominal pain, mean (11-point NRS scale; SD)	CSBMs/week, mean (SD)	Patients assigned to active drug, dosage, schedule, and duration of therapy	Outcomes
Rao et al. (2012)	United states and Canada; 118	Rome II criteria	800 (90.5)	EG: 43.3 (19–81) CG:43.7 (18–84)	EG: 5.7 (1.7) CG: 5.6 (1.7)	EG: 0.2 (0.5) CG: 0.2 (0.5)	405 patients received linaclotide 290 μg od for 12 weeks	①②③⑤⑥⑦
Chey et al. (2012)	United states; 102	Rome II criteria	804 (89.6)	EG: 44.6 (19–82) CG:44.0 (18–87)	EG: 5.6 (1.7) CG: 5.5 (1.7)	EG: 0.2 (0.4) CG: 0.2 (0.4)	401 patients received linaclotide 290 μg od for 26 weeks	①②③⑤⑥⑦
Yang et al. (2018)	China, United states, Canada Australia, New Zealand	Rome III criteria	839 (82.0)	EG: 41.0 (18–77) CG:41.3 (18–80)	EG: 5.2 (1.5) CG: 5.2 (1.5)	EG: 0.3 (0.6) CG: 0.2 (0.5)	417 patients received linaclotide 290 μg od for 12 weeks	①④⑤⑥⑦
Chang et al. (2021)	United states; 78	Rome III criteria	614 (80.8)	EG: 46.5 (19–85) CG:46.8 (18–79)	EG: 6.22 (1.69) CG: 6.26 (1.66)	EG: 0.27 (0.51) CG: 0.26 (0.53)	306 patients received linaclotide 290 μg od for 12 weeks	①②③④⑤⑤⑥⑦
Johnston et al. (2010)	United states and Canada; 92	Rome II criteria	169 (91.7)	EG: 46.0 (21–72) CG:44.3 (21–65)	EG: unclear CG: unclear	EG: 0.2 (0.5) CG: 0.3 (0.5)	84 patients received linaclotide 290 μg od for 12 weeks	④⑥⑦
Chey et al. (2021)	United states; 71	Rome III criteria	132 (80.3)	EG: 44.1 (14.4) CG:45.4 (14.7)	EG: 6.18 (1.77) CG: 6.41 (1.85)	EG: 0.34 (0.61) CG: 0.29 (0.56)	66 patients received linaclotide 290 μg od for 12 weeks	①②③⑤⑥⑦
NCT02387359	North America; 130	Rome III criteria	1054 (76.4)	EG (3 mg):43.0 (13.8) EG (6 mg):43.2 (13.3) CG: 43.0 (13.7)	EG (3 mg): 5.9 (1.7) EG (6 mg): 6.0 (1.8) CG: 6.1 (1.8)	EG (3 mg): 0.2 (0.5) EG (6 mg): 0.3 (0.5) CG: 0.2 (0.4)	351 and 349 patients received plecanatide 3 and 6 mg od, respectively, for 12 weeks	①②③④⑤⑤⑥⑦
NCT02493452	North America; 140	Rome III criteria	1135 (71.8)	EG (3 mg):44.0 (14.6) EG (6 mg):43.1 (14.2) CG:44.8 (14.7)	EG (3 mg): 6.6 (1.6) EG (6 mg): 6.5 (1.7) CG: 6.4 (1.6)	EG (3 mg): 0.3 (0.5) EG (6 mg): 0.3 (0.5) CG: 0.2 (0.5)	377 and 379 patients received plecanatide 3 and 6 mg od, respectively, for 12 weeks	①②③④⑤⑤⑥⑦
Miner et al. (2014)	United states; 99	Rome III criteria	171 (81.3)	EG: 45.4 (12.02) CG:47.2 (12.89)	EG: unclear CG: unclear	EG: unclear CG: unclear	86 patients received plecanatide 3 mg od for 12 weeks	①⑤⑥⑦

EG, experimental group; CG, control group; (①): FDA endpoint (each week, ≥30% decrease in abdominal pain + an increase ≥1 CSBM from baseline for at least 6/12 weeks); (②):abdominal pain responders (patients with ≥30% decrease in abdominal pain)for at least 6/12 weeks; (③):CSBM responders (patients w/CSBM rate increase ≥1 per week)for at least 6/12 weeks; (④):SBM ≤24 h after first dose; (⑤):change from baseline over the 12-week Treatment Period in abdominal pain; (⑥):change from baseline over the 12-week Treatment Period in CSBM Frequency Rate (CSBMs/Week); (⑦):patients with diarrhea over the 12-week Treatment Period.

### 3.3 Risk of bias in trials

**FIGURE 2:**
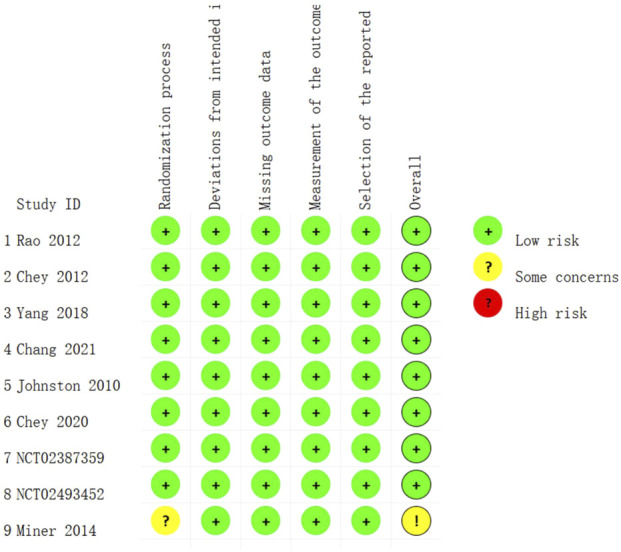
Risk of bias summary: review authors’ judgment of each risk of bias item for each included trial.

**FIGURE 3:**
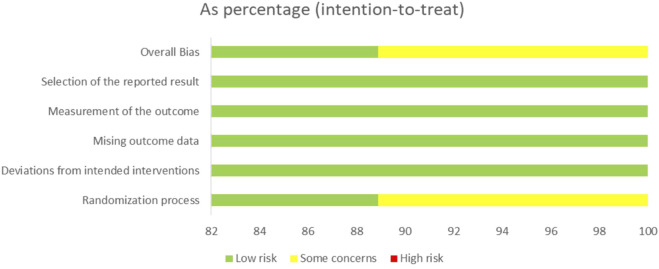
Risk of bias plot: Review authors’ judgment for each risk of bias item, expressed as a percentage across all included trials.

The risk of bias graph for each included trial is shown in Figure 2, and the percentages for each trial are shown in Figure 3. All nine trials employed randomization principles, with eight utilizing IVRS or IWRS (Interactive Voice/Web Response Systems) for random sequence generation and allocation concealment (Brenner et al., 2018; Rao et al., 2012; Chey et al., 2012; Yang et al., 2018; Chang et al., 2021; Johnston et al., 2010; Chey et al., 2021). The remaining trial mentioned using IVRS for allocation concealment, but did not detail the randomization process. All studies employed a double-blind design, fully reported the integrity of outcome data, and demonstrated minimal selective reporting bias with no other biases present.

### 3.4 Results of the meta-analysis

#### 3.4.1 Primary outcome

##### 3.4.1.1 FDA responders endpoint

**FIGURE 4:**
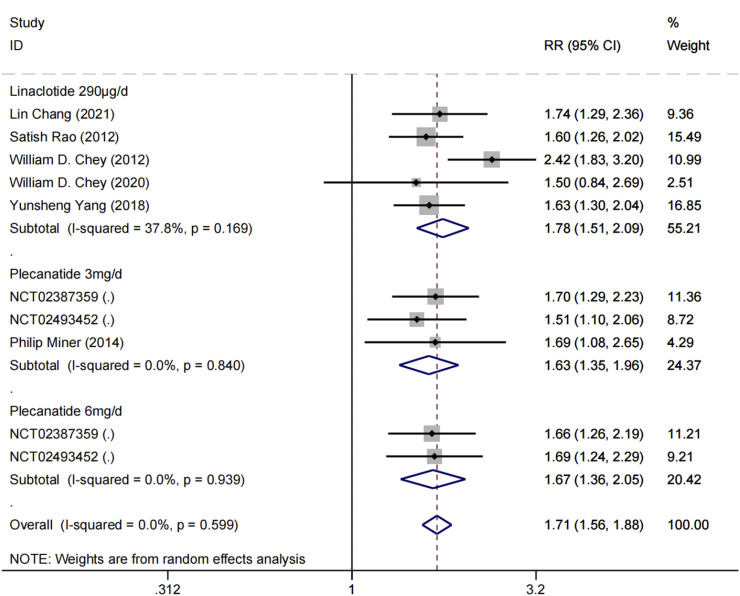
Forest plots for analysis of efficacy based on FDA responder endpoint in treating IBS-C with linaclotide or plecanatide.

**FIGURE 5:**
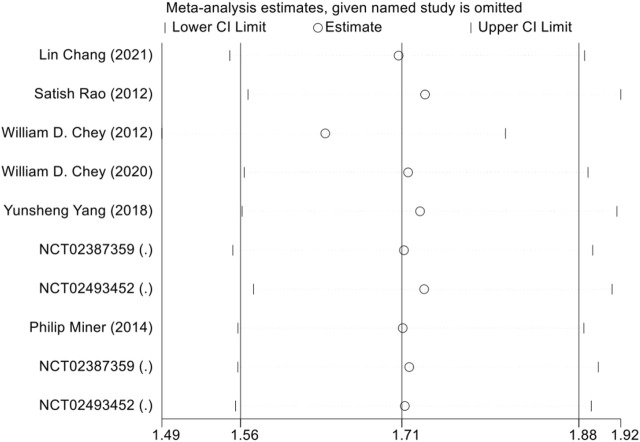
Sensitivity analysis of FDA responder endpoint.

A total of 8 trials (Brenner et al., 2018; Rao et al., 2012; Chey et al., 2012; Yang et al., 2018; Chang et al., 2021; Chey et al., 2021; Miner et al., 2014) reported the proportion of patients achieving the FDA composite endpoint, involving 5,549 patients. A random-effects meta-analysis indicated that GCC agonists were more likely than placebo to achieve the FDA composite endpoint for IBS-C (overall RR = 1.71, 95% CI [1.56, 1.88], Z = 11.39, P for overall effect <0.0001), with no evidence of heterogeneity across studies (P for heterogeneity = 0.599, I^2^ = 0%). Subgroup analyses indicated that linaclotide 290 μg (RR = 1.78, 95% CI [1.51, 2.09], I^2^ = 37.8%); plecanatide 3 mg (RR = 1.63, 95% CI [1.35, 1.96], I^2^ = 0%); plecanatide 6 mg (RR = 1.67, 95% CI [1.36, 2.05], I^2^ = 0%) (Figure 4). Sensitivity analysis indicates that excluding any single study does not affect the stability of the trial results (Figure 5).

#### 3.4.2 Secondary outcomes

##### 3.4.2.1 Abdominal pain responders

A total of 6 trials (Brenner et al., 2018; Rao et al., 2012; Chey et al., 2012; Chang et al., 2021; Chey et al., 2021) reported the proportion of patients with abdominal pain who responded among 4,539 patients. A random-effects meta-analysis revealed that patients treated with GCC agonists had a higher proportion (≥30% reduction in abdominal pain for at least 6 weeks) during the 12-week treatment period than the control group (overall RR = 1.39, 95% CI [1.29, 1.49], Z = 8.91, P for overall effect <0.0001), with no evidence of heterogeneity across studies (P for heterogeneity = 0.716, I^2^ = 0%). Subgroup analyses indicated that linaclotide 290 μg (RR = 1.39, 95% CI [1.23, 1.56], I^2^ = 23.4%); plecanatide 3 mg (RR = 1.35, 95% CI [1.16, 1.57], I^2^ = 0%); plecanatide 6 mg (RR = 1.43, 95% CI [1.24, 1.66], I^2^ = 0%) (Supplementary Image 1).

##### 3.4.2.2 CSBM responders

A total of 6 trials (Brenner et al., 2018; Rao et al., 2012; Chey et al., 2012; Chang et al., 2021; Chey et al., 2021) reported the proportion of CSBM responders among 4,539 patients. A meta-analysis using a random-effect model showed an increase in CSBM of ≥1 week compared to baseline for at least 6 weeks during the 12-week treatment period, relative to the control group (overall RR = 1.49, 95% CI [1.32, 1.68], Z = 6.43, P for overall effect <0.0001), with substantial heterogeneity across studies (P for heterogeneity = 0.009, I^2^ = 62.9%). Subgroup analyses indicated that linaclotide 290 μg (RR = 1.70, 95% CI [1.45, 2.00], I^2^ = 49.5%); plecanatide 3 mg (RR = 1.31, 95% CI [1.14, 1.50], I^2^ = 0%); plecanatide 6 mg (RR = 1.33, 95% CI [1.16, 1.53], I^2^ = 0%) (Supplementary Image 1).

##### 3.4.2.3 SBM within 24 h after first dose

A total of 5 trials (Brenner et al., 2018; Yang et al., 2018; Chang et al., 2021; Johnston et al., 2010) involving 3,811 patients were included. A random-effects meta-analysis revealed that patients treated with GCC agonists demonstrated a higher proportion of spontaneous bowel movements within 24 h of the first dose (overall RR = 1.52, 95% CI [1.41, 1.63], Z = 11.14, P for overall effect <0.0001), with no evidence of heterogeneity across studies (P for heterogeneity = 0.422, I^2^ = 0.2%). Subgroup analyses indicated that linaclotide 290 μg (RR = 1.69, 95% CI [1.50, 1.90], I^2^ = 0%); plecanatide 3 mg (RR = 1.38, 95% CI [1.21, 1.58], I^2^ = 0%); plecanatide 6 mg (RR = 1.45, 95% CI [1.28, 1.66], I^2^ = 0%) (Supplementary Image 1).

##### 3.4.2.4 Abdominal pain (change from baseline)

A total of 8 trials (Brenner et al., 2018; Rao et al., 2012; Chey et al., 2012; Yang et al., 2018; Chang et al., 2021; Chey et al., 2021; Miner et al., 2014) involving 5,546 patients reported the severity of abdominal pain. A random-effects meta-analysis revealed that GCC agonists effectively improved abdominal pain compared to placebo (overall WMD = −0.62, 95% CI [-0.72, −0.51], Z = 11.74, P for overall effect <0.0001), with no evidence of heterogeneity across studies (P for heterogeneity = 0.518, I^2^ = 0%). Subgroup analyses indicated that linaclotide 290 μg (WMD = −0.70, 95% CI [-0.84, −0.56], I^2^ = 0%); plecanatide 3 mg (WMD = −0.48, 95% CI [-0.68, −0.27], I^2^ = 0%); plecanatide 6 mg (WMD = −0.55, 95% CI [-0.78, −0.32], I^2^ = 0%) (Supplementary Image 1).

##### 3.4.2.5 CSBM frequency (change from baseline)

A total of 9 trials (Brenner et al., 2018; Rao et al., 2012; Chey et al., 2012; Yang et al., 2018; Chang et al., 2021; Johnston et al., 2010; Chey et al., 2021; Miner et al., 2014) involving 5,713 patients reported CSBM frequency. A meta-analysis using a random-effects model demonstrated that GCC agonists effectively increased the number of complete spontaneous bowel movements compared with placebo (overall WMD = 1.10, 95% CI [0.78, 1.41], Z = 6.83, P for overall effect <0.0001), with substantial heterogeneity across studies (P for heterogeneity = 0.000, I^2^ = 83.4%). Subgroup analyses indicated that linaclotide 290 μg (WMD = 1.43, 95% CI [1.11, 1.74], I^2^ = 57.7%); plecanatide 3 mg (WMD = 0.71, 95% CI [0.28, 1.15], I^2^ = 70.8%); plecanatide 6 mg (WMD = 0.68, 95% CI [0.41, 0.96], I^2^ = 20.6%) (Supplementary Image 1).

#### 3.4.3 Adverse events

**FIGURE 6:**
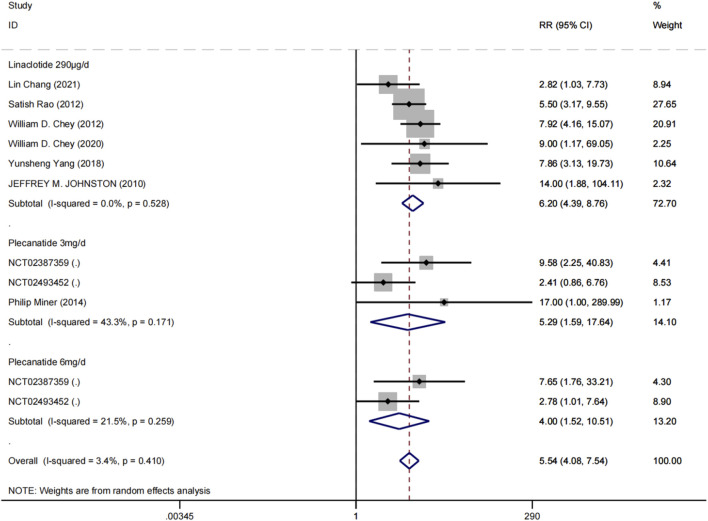
Forest plots for analysis of safety based on diarrhea rates in treating IBS-C with linaclotide or plecanatide.

A total of 9 trials (Brenner et al., 2018; Rao et al., 2012; Chey et al., 2012; Yang et al., 2018; Chang et al., 2021; Johnston et al., 2010; Chey et al., 2021; Miner et al., 2014) involving 5,712 patients reported the incidence of diarrhea. A random-effects meta-analysis revealed that GCC agonists were more likely to be associated with diarrhea compared to placebo (overall RR = 5.54, 95% CI [4.08, 7.54], Z = 10.93, P for overall effect <0.0001), with no evidence of heterogeneity across studies (P for heterogeneity = 0.410, I^2^ = 3.4%). Subgroup analyses indicated that linaclotide 290 μg (RR = 6.20, 95% CI [4.39, 8.76], I^2^ = 0%); plecanatide 3 mg (RR = 5.29, 95% CI [1.59, 17.64], I^2^ = 43.3%); plecanatide 6 mg (RR = 4.00, 95% CI [1.52, 10.51], I^2^ = 21.5%) (Figure 6).

### 3.5 Certainty of evidence

The GRADE results are presented in the Supplementary Appendix Table S3. Except for the evidence on “mean change in CSBM from baseline,” which is rated as “moderate,” the evidence for all other outcome measures is rated as “high.“The downgrade was due to higher heterogeneity.

## 4 Discussion

This study focuses on the efficacy and safety of GCC agonists for the treatment of IBS-C. By incorporating recent trials, including those by Chang (2021), Chey (2020), and others, it provides an update to previous meta-analyses on similar topics by Shah et al. (2018); Black et al. (2018). We methodically evaluated the efficacy of GCC agonists in ameliorating the primary symptoms of IBS-C by incorporating nine high-caliber randomized controlled clinical trials. The results indicated that linaclotide and plecanatide significantly surpassed placebo in the primary objective (FDA composite endpoint) and other secondary endpoints, exhibiting very consistent performance across these metrics.

### 4.1 Efficacy of the drug

The study indicated that GCC agonists exhibited an overall effect size of 1.71 on the key outcome measure—the FDA composite endpoint—with no heterogeneity (I^2^ = 0%). The results reveal that patients in the therapy group exhibited a markedly greater probability of attaining dual symptom amelioration. The strong consistency of these data highlights the dependable effectiveness of GCC agonists as a category of medications for the treatment of IBS-C. Designating the FDA composite endpoint as the “gold standard” accurately reflects the complex symptomatology of IBS-C, hence augmenting the clinical relevance of this study’s results. Physicians may base their selections on medicine availability, health insurance policies, cost-effectiveness, and specific patient preferences, without excessive apprehension of substantial disparities in efficacy.

A comprehensive examination of secondary endpoints further supports the dual pharmacological mechanism of GCC agonists. Outcomes associated with abdominal pain: The medications exhibited reliable effectiveness in mitigating abdominal pain, with an overall response rate enhancement of 39% and an average decrease in pain scores of 0.62. This verifies that GCC medications specifically target intestinal pain nerve terminals by increasing extracellular cGMP levels, thereby diminishing visceral hypersensitivity and alleviating abdominal pain (Cas et al., 2013; Grundy et al., 2018). Outcomes associated with constipation: The overall enhancement in CSBM response rate attained 49%, with linaclotide exhibiting a more pronounced effect (70% compared to 31%–33% for plecanatide). The total increase in CSBM frequency was 1.10 times per week; however, the enhancement with linaclotide (1.43 times/week) was substantially superior to that with plecanatide (0.68–0.71 times/week), indicating its efficacy in stimulating intestinal secretion and motility. The proportion obtaining SBM within 24 h following the initial dose increased by 52% overall. Linaclotide (69%) had a quick onset of action, providing substantial therapeutic benefit for patients in need of immediate symptom alleviation.

### 4.2 Safety of the drug

Diarrhea, as a class effect of GCC agonists, was considerably elevated compared to placebo in all treatment groups (overall RR = 5.54), directly resulting from the drug’s prosecretory action. The risk of diarrhea associated with linaclotide (RR = 6.20) is quantitatively greater than that with plecanatide; however, this disparity should be approached with caution. Shah et al. (49) observed considerable discrepancies in the criteria of diarrhea among clinical trials: the plecanatide study utilized a more stringent definition, documenting only “bothersome or treatment-requiring diarrhea,” whereas the linaclotide trial implemented conventional adverse event collection techniques. The definitional discrepancies certainly constitute a significant factor influencing the variance in reported incidence rates. Nonetheless, the overall incidence of diarrhea in this study did not surpass 10%, with the majority of cases being mild to moderate in severity and self-limiting, suggesting that this adverse reaction can be effectively managed through patient education and dosage modifications (including temporary cessation or alternating day dosing). In clinical practice, patients must get comprehensive education and communication at the initiation of treatment to improve adherence.

### 4.3 Difference analysis

Although no statistically significant differences were observed among the three GCC agonist regimens, the point estimates consistently favored linaclotide 290 µg over plecanatide 3 mg and 6 mg across multiple efficacy outcomes. For instance, linaclotide demonstrated a higher relative risk for achieving the FDA composite endpoint (RR = 1.78, 95% CI: 1.51–2.09) compared to plecanatide 3 mg (RR = 1.63, 95% CI: 1.35–1.96) and plecanatide 6 mg (RR = 1.67, 95% CI: 1.36–2.05). Similar trends were observed in secondary endpoints, such as the proportion of CSBM responders (linaclotide: RR = 1.70, 95% CI: 1.45–2.00; plecanatide 3 mg: RR = 1.31, 95% CI: 1.14–1.50; plecanatide 6 mg: RR = 1.33, 95% CI: 1.16–1.53) and the mean change in CSBM frequency from baseline (linaclotide: WMD = 1.43, 95% CI: 1.11–1.74; plecanatide 3 mg: WMD = 0.71, 95% CI: 0.28–1.15; plecanatide 6 mg: WMD = 0.68, 95% CI: 0.41–0.96).

Despite these numerical advantages, the lack of statistical significance may be attributed to several factors:

#### 4.3.1 Sample size and power

Although the total sample size was substantial (N = 5,718), the number of trials for plecanatide was limited (only 3 trials), which may have reduced the statistical power to detect modest between-drug differences. This is particularly relevant for outcomes with wide confidence intervals, such as CSBM frequency change for plecanatide 3 mg (WMD = 0.71, 95% CI: 0.28–1.15), indicating greater uncertainty.

#### 4.3.2 Heterogeneity in trial design and endpoint definitions

While the FDA composite endpoint was consistently reported, subtle differences in how secondary endpoints were defined or measured across trials may have introduced variability. For example, the definition of diarrhea varied between linaclotide and plecanatide trials, with the latter employing a stricter criterion (only “bothersome or treatment-requiring” diarrhea), which may have influenced safety reporting and indirectly affected tolerability and adherence.

#### 4.3.3 Regional and baseline differences

Studies were conducted across diverse geographic regions (e.g., North America, China, Australia), and baseline symptom severity varied slightly (e.g., abdominal pain scores ranged from 5.2 to 6.6). These factors may have contributed to within-drug variability, potentially masking true between-drug differences.

#### 4.3.4 pH-dependent

Plecanatide, an analog of human uroguanylin, demonstrates considerable pH dependence in its receptor binding. It demonstrates maximal affinity for GCC receptors in acidic conditions, specifically in the duodenum and proximal jejunum. As chyme progresses into the mid-to-distal gut, its neutral pH results in a reduced binding capacity (Brancale et al., 2017). This indicates that the effects of plecanatide may be more pronounced in the proximal small intestine. Linaclotide is a synthetic peptide whose structure diverges from that of uridylate, as it does not contain a sulfated tyrosine residue. This enables its binding to the GCC receptor to remain pH-independent, preserving high affinity throughout the extensive pH spectrum of the gastrointestinal tract, from the proximal small intestine to the colon (Busby and Ortiz, 2014). This attribute expands its operational range, facilitating the ongoing stimulation of fluid secretion and peristalsis in the distal small intestine and colon, which are essential for stool production and excretion. The variations in modes of action may explain the discrepancies in diarrhea rates between the two medications (Miner et al., 2017).

#### 4.3.5 Intracellular cGMP

Linaclotide’s highly effective receptor binding throughout a larger tract of the gut may facilitate elevated or prolonged cGMP signaling in a broader array of intestinal epithelial cells. Enhanced cGMP signaling promotes elevated chloride and bicarbonate secretion, resulting in a more significant secretagogue action that more efficiently softens stool and expedites colonic transit. Research suggests (Busby et al., 2013) that the active constituents of linaclotide are detectable in faeces, implying that it may preserve structural integrity and functional efficacy during gastrointestinal transit. This stability may extend its duration of action, resulting in more prolonged secretagogue and prokinetic effects.

#### 4.3.6 Extracellular cGMP

While both medicines alleviate stomach pain by blocking nociceptive pathways via elevated extracellular cGMP, linaclotide’s superior effectiveness in ameliorating constipation may indirectly enhance abdominal pain relief. More efficiently alleviates constipation and bloating by diminishing the physical stimulation of mechanosensitive nerves resulting from faecal retention and intestinal distension; expedited intestinal transit may mitigate the buildup of metabolic byproducts, such as fermentation-derived gases and short-chain fatty acids, in the gut, which can activate chemosensitive receptors and induce pain (Barbara et al., 2021). The alleviation of stomach pain with linaclotide may stem from two factors: its analgesic properties and the secondary advantage of its enhanced laxative impact.

#### 4.3.7 Metabolic stability

The pharmacokinetic characteristics of the two drugs vary. All of these substances exert local effects in the colon, exhibiting minimal systemic bioavailability that is insignificant. Nonetheless, their metabolic stability in the gastrointestinal tract may fluctuate, influencing the effective concentration and duration of presence at the site of action. Linaclotide may demonstrate enhanced stability against intestinal proteases, hence preserving elevated effective concentrations upon arrival in distal locations such as the colon and sustaining therapeutic effects. Additional investigation is required to examine this concept.

### 4.4 Limitations

This study concurrently presents numerous limitations: (a) The limited number of randomized controlled trials ultimately included, particularly those involving plecanatide, may have somewhat diminished the statistical power for certain outcome measures, especially in drug efficacy comparisons; (b) The inclusion criteria primarily relied on Rome II/III standards, and its relevance to the Rome IV criteria population requires validation; (c) Due to constraints in the original data, it is impossible to investigate efficacy differences among specific subgroups (e.g., varying genders, ages, baseline symptom severity) based on individual patient data; (d) All drug comparisons are founded on indirect evidence; future head-to-head randomized controlled trials are essential to furnish the most robust direct comparative evidence.

## 5 Conclusion

This meta-analysis confirms that GCC agonists (linaclotide and plecanatide) significantly improve overall symptoms in patients with IBS-C. They demonstrate superior efficacy to placebo in achieving the FDA composite endpoint, alleviating abdominal pain, and improving bowel movements, with highly consistent therapeutic effects. Although the differences between different drugs and doses were not statistically significant, supporting their “class effect,” linaclotide showed a numerically superior trend, which may be related to its pH-independent mechanism of action. Although the risk of diarrhea is significantly increased with this class of drugs, adverse reactions are mostly mild to moderate and can be managed through appropriate control measures. In clinical practice, individualized selection should be based on confirming equivalent efficacy, while also considering treatment trends, safety, drug accessibility, and patient preferences. Future head-to-head studies are needed to validate further differences between drugs and their applicability in populations meeting Rome IV criteria.

## Data Availability

The original contributions presented in the study are included in the article/Supplementary Material, further inquiries can be directed to the corresponding authors.

## Supplementary material

SUPPLEMENTARY FIGURE S1 Secondary forest plot. **(A)** abdominal pain responders; **(B)** CSBM responders; **(C)** SBM within 24 h after first dose; **(D)** abdominal pain (change from baseline); **(E)** CSBM frequency (change from baseline).
