# Supplementary figures and images for: Efficacy and safety of Guanylyl cyclase C agonists (linaclotide and plecanatide) in patients with irritable bowel syndrome with constipation: a systematic review and meta-analysis of randomized controlled trials
Source: Front Pharmacol. 2026 Apr 10;17:1761301. doi: 10.3389/fphar.2026.1761301 (PMC13106150; doi:10.3389/fphar.2026.1761301)

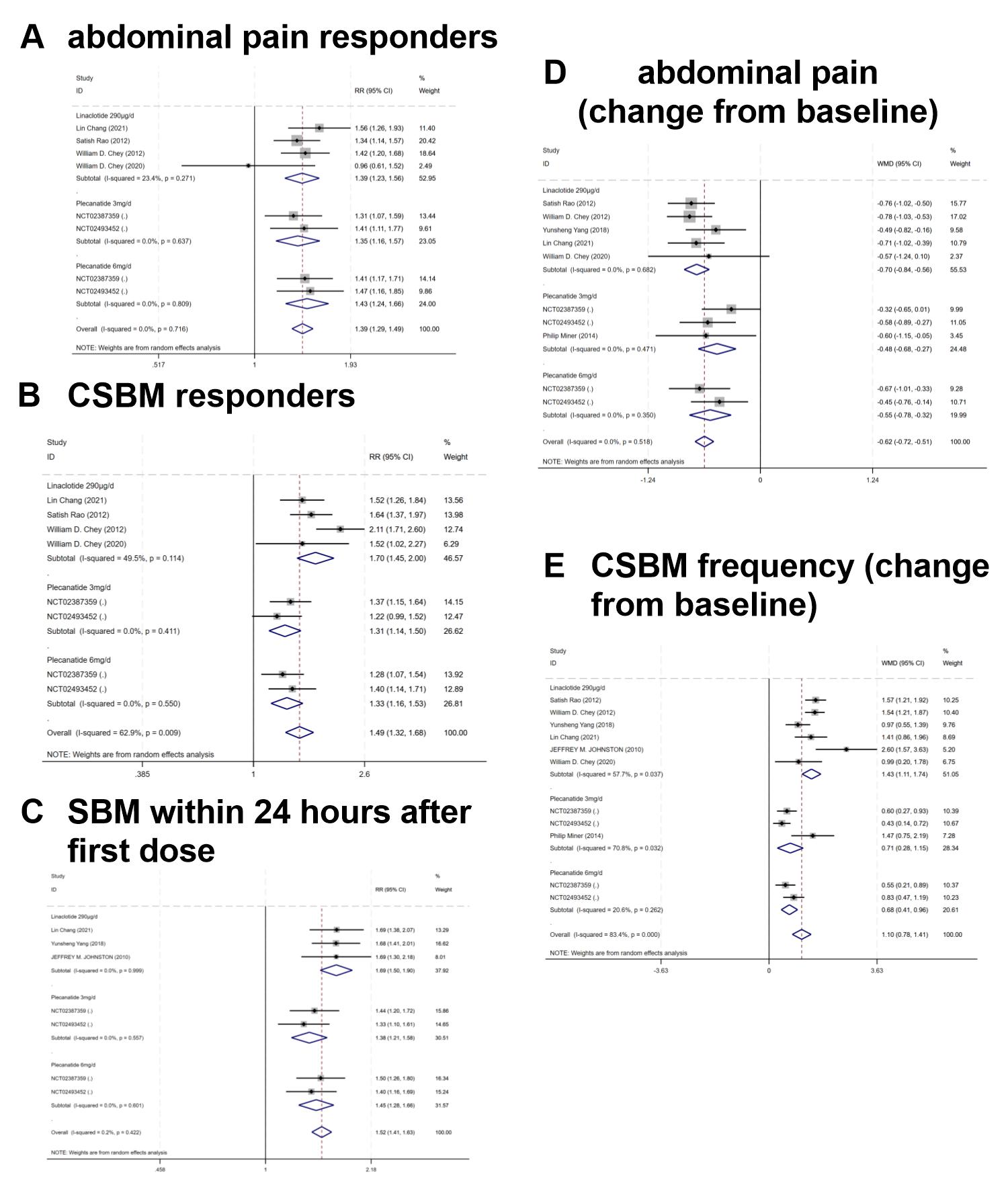

Supplement: Supplementary file 2 [file Image1.jpeg]
