# Supplementary material for: Efficacy and safety of Guanylyl cyclase C agonists (linaclotide and plecanatide) in patients with irritable bowel syndrome with constipation: a systematic review and meta-analysis of randomized controlled trials
Source: Front Pharmacol. 2026 Apr 10;17:1761301. doi: 10.3389/fphar.2026.1761301 (PMC13106150; doi:10.3389/fphar.2026.1761301)
Supplement: Supplementary file 3 [file Table2.docx]

Efficacy and Safety of Guanylyl Cyclase C Agonists (Linaclotide and Plecanatide) in patients with irritable bowel syndrome with constipation: a systematic review and meta-analysis of randomized controlled trials

**Pubmed：**

| #16 |  |  | Search: ****(((("Irritable Bowel Syndrome"[Mesh]) OR (((((((((Irritable Bowel Syndromes[Title/Abstract]) OR (Syndrome, Irritable Bowel[Title/Abstract])) OR (Syndromes, Irritable Bowel[Title/Abstract])) OR (Colitis, Mucous[Title/Abstract])) OR (Colitides, Mucous[Title/Abstract])) OR (Mucous Colitides[Title/Abstract])) OR (Mucous Colitis[Title/Abstract])) OR (Colon, Irritable[Title/Abstract])) OR (Irritable Colon[Title/Abstract]))) AND (("Constipation"[Mesh]) OR ((Colonic Inertia[Title/Abstract]) OR (Dyschezia[Title/Abstract])))) AND (((((("Guanylyl Cyclase C Agonists"[Mesh]) OR (Guanylate Cyclase C Agonists[Title/Abstract])) OR ("linaclotide" [Supplementary Concept])) OR ((((((MD-1100 acetate[Title/Abstract]) OR (Linaclotide Acetate[Title/Abstract])) OR (Linzess[Title/Abstract])) OR (MD-1100[Title/Abstract])) OR (ASP-0456[Title/Abstract])) OR (ASP0456[Title/Abstract]))) OR ("plecanatide" [Supplementary Concept])) OR ((Trulance[Title/Abstract]) OR (SP-304[Title/Abstract])))) AND (randomized controlled trial[Publication Type] OR randomized[Title/Abstract] OR placebo[Title/Abstract])**** | [39](https://pubmed.ncbi.nlm.nih.gov/?term=(((("Irritable+Bowel+Syndrome"[Mesh])+OR+(((((((((Irritable+Bowel+Syndromes[Title/Abstract])+OR+(Syndrome,+Irritable+Bowel[Title/Abstract]))+OR+(Syndromes,+Irritable+Bowel[Title/Abstract]))+OR+(Colitis,+Mucous[Title/Abstract]))+OR+(Colitides,+Mucous[Title/Abstract]))+OR+(Mucous+Colitides[Title/Abstract]))+OR+(Mucous+Colitis[Title/Abstract]))+OR+(Colon,+Irritable[Title/Abstract]))+OR+(Irritable+Colon[Title/Abstract])))+AND+(("Constipation"[Mesh])+OR+((Colonic+Inertia[Title/Abstract])+OR+(Dyschezia[Title/Abstract]))))+AND+(((((("Guanylyl+Cyclase+C+Agonists"[Mesh])+OR+(Guanylate+Cyclase+C+Agonists[Title/Abstract]))+OR+("linaclotide"+[Supplementary+Concept]))+OR+((((((MD-1100+acetate[Title/Abstract])+OR+(Linaclotide+Acetate[Title/Abstract]))+OR+(Linzess[Title/Abstract]))+OR+(MD-1100[Title/Abstract]))+OR+(ASP-0456[Title/Abstract]))+OR+(ASP0456[Title/Abstract])))+OR+("plecanatide"+[Supplementary+Concept]))+OR+((Trulance[Title/Abstract])+OR+(SP-304[Title/Abstract]))))+AND+(randomized+controlled+trial[Publication+Type]+OR+randomized[Title/Abstract]+OR+placebo[Title/Abstract])&sort=) | 05:45:46 |
| --- | --- | --- | --- | --- | --- |
| #15 |  |  | Search: ****randomized controlled trial[Publication Type] OR randomized[Title/Abstract] OR placebo[Title/Abstract]**** | [1,145,455](https://pubmed.ncbi.nlm.nih.gov/?term=randomized+controlled+trial[Publication+Type]+OR%0D%0Arandomized[Title/Abstract]+OR%0D%0Aplacebo[Title/Abstract]&sort=) | 05:42:50 |
| #14 |  |  | Search: ****((((("Guanylyl Cyclase C Agonists"[Mesh]) OR (Guanylate Cyclase C Agonists[Title/Abstract])) OR ("linaclotide" [Supplementary Concept])) OR ((((((MD-1100 acetate[Title/Abstract]) OR (Linaclotide Acetate[Title/Abstract])) OR (Linzess[Title/Abstract])) OR (MD-1100[Title/Abstract])) OR (ASP-0456[Title/Abstract])) OR (ASP0456[Title/Abstract]))) OR ("plecanatide" [Supplementary Concept])) OR ((Trulance[Title/Abstract]) OR (SP-304[Title/Abstract]))**** | [280](https://pubmed.ncbi.nlm.nih.gov/?term=((((("Guanylyl+Cyclase+C+Agonists"[Mesh])+OR+(Guanylate+Cyclase+C+Agonists[Title/Abstract]))+OR+("linaclotide"+[Supplementary+Concept]))+OR+((((((MD-1100+acetate[Title/Abstract])+OR+(Linaclotide+Acetate[Title/Abstract]))+OR+(Linzess[Title/Abstract]))+OR+(MD-1100[Title/Abstract]))+OR+(ASP-0456[Title/Abstract]))+OR+(ASP0456[Title/Abstract])))+OR+("plecanatide"+[Supplementary+Concept]))+OR+((Trulance[Title/Abstract])+OR+(SP-304[Title/Abstract]))&sort=) | 05:38:25 |
| #13 |  |  | Search: ****(Trulance[Title/Abstract]) OR (SP-304[Title/Abstract])**** | [17](https://pubmed.ncbi.nlm.nih.gov/?term=(Trulance[Title/Abstract])+OR+(SP-304[Title/Abstract])&sort=) | 05:33:44 |
| #12 |  |  | Search: ****"plecanatide" [Supplementary Concept]**** Sort by: ****Most Recent**** | [43](https://pubmed.ncbi.nlm.nih.gov/?sort=date&term="plecanatide"+[Supplementary+Concept]) | 05:32:33 |
| #11 |  |  | Search: ****(((((MD-1100 acetate[Title/Abstract]) OR (Linaclotide Acetate[Title/Abstract])) OR (Linzess[Title/Abstract])) OR (MD-1100[Title/Abstract])) OR (ASP-0456[Title/Abstract])) OR (ASP0456[Title/Abstract])**** | [30](https://pubmed.ncbi.nlm.nih.gov/?term=(((((MD-1100+acetate[Title/Abstract])+OR+(Linaclotide+Acetate[Title/Abstract]))+OR+(Linzess[Title/Abstract]))+OR+(MD-1100[Title/Abstract]))+OR+(ASP-0456[Title/Abstract]))+OR+(ASP0456[Title/Abstract])&sort=) | 05:29:27 |
| #10 |  |  | Search: ****"linaclotide" [Supplementary Concept]**** Sort by: ****Most Recent**** | [199](https://pubmed.ncbi.nlm.nih.gov/?sort=date&term="linaclotide"+[Supplementary+Concept]) | 05:27:24 |
| #9 |  |  | Search: ****Guanylate Cyclase C Agonists[Title/Abstract]**** | [18](https://pubmed.ncbi.nlm.nih.gov/?term=Guanylate+Cyclase+C+Agonists[Title/Abstract]&sort=) | 05:26:26 |
| #8 |  |  | Search: ****"Guanylyl Cyclase C Agonists"[Mesh]**** Sort by: ****Most Recent**** | [70](https://pubmed.ncbi.nlm.nih.gov/?sort=date&term="Guanylyl+Cyclase+C+Agonists"[Mesh]) | 05:25:53 |
| #7 |  |  | Search: ****(("Irritable Bowel Syndrome"[Mesh]) OR (((((((((Irritable Bowel Syndromes[Title/Abstract]) OR (Syndrome, Irritable Bowel[Title/Abstract])) OR (Syndromes, Irritable Bowel[Title/Abstract])) OR (Colitis, Mucous[Title/Abstract])) OR (Colitides, Mucous[Title/Abstract])) OR (Mucous Colitides[Title/Abstract])) OR (Mucous Colitis[Title/Abstract])) OR (Colon, Irritable[Title/Abstract])) OR (Irritable Colon[Title/Abstract]))) AND (("Constipation"[Mesh]) OR ((Colonic Inertia[Title/Abstract]) OR (Dyschezia[Title/Abstract])))**** | [1,131](https://pubmed.ncbi.nlm.nih.gov/?term=(("Irritable+Bowel+Syndrome"[Mesh])+OR+(((((((((Irritable+Bowel+Syndromes[Title/Abstract])+OR+(Syndrome,+Irritable+Bowel[Title/Abstract]))+OR+(Syndromes,+Irritable+Bowel[Title/Abstract]))+OR+(Colitis,+Mucous[Title/Abstract]))+OR+(Colitides,+Mucous[Title/Abstract]))+OR+(Mucous+Colitides[Title/Abstract]))+OR+(Mucous+Colitis[Title/Abstract]))+OR+(Colon,+Irritable[Title/Abstract]))+OR+(Irritable+Colon[Title/Abstract])))+AND+(("Constipation"[Mesh])+OR+((Colonic+Inertia[Title/Abstract])+OR+(Dyschezia[Title/Abstract])))&sort=) | 05:24:13 |
| #6 |  |  | Search: ****("Constipation"[Mesh]) OR ((Colonic Inertia[Title/Abstract]) OR (Dyschezia[Title/Abstract]))**** | [17,778](https://pubmed.ncbi.nlm.nih.gov/?term=("Constipation"[Mesh])+OR+((Colonic+Inertia[Title/Abstract])+OR+(Dyschezia[Title/Abstract]))&sort=) | 05:23:51 |
| #5 |  |  | Search: ****(Colonic Inertia[Title/Abstract]) OR (Dyschezia[Title/Abstract])**** | [706](https://pubmed.ncbi.nlm.nih.gov/?term=(Colonic+Inertia[Title/Abstract])+OR+(Dyschezia[Title/Abstract])&sort=) | 05:23:37 |
| #4 |  |  | Search: ****"Constipation"[Mesh]**** Sort by: ****Most Recent**** | [17,329](https://pubmed.ncbi.nlm.nih.gov/?sort=date&term="Constipation"[Mesh]) | 05:22:34 |
| #3 |  |  | Search: ****("Irritable Bowel Syndrome"[Mesh]) OR (((((((((Irritable Bowel Syndromes[Title/Abstract]) OR (Syndrome, Irritable Bowel[Title/Abstract])) OR (Syndromes, Irritable Bowel[Title/Abstract])) OR (Colitis, Mucous[Title/Abstract])) OR (Colitides, Mucous[Title/Abstract])) OR (Mucous Colitides[Title/Abstract])) OR (Mucous Colitis[Title/Abstract])) OR (Colon, Irritable[Title/Abstract])) OR (Irritable Colon[Title/Abstract]))**** | [10,942](https://pubmed.ncbi.nlm.nih.gov/?term=("Irritable+Bowel+Syndrome"[Mesh])+OR+(((((((((Irritable+Bowel+Syndromes[Title/Abstract])+OR+(Syndrome,+Irritable+Bowel[Title/Abstract]))+OR+(Syndromes,+Irritable+Bowel[Title/Abstract]))+OR+(Colitis,+Mucous[Title/Abstract]))+OR+(Colitides,+Mucous[Title/Abstract]))+OR+(Mucous+Colitides[Title/Abstract]))+OR+(Mucous+Colitis[Title/Abstract]))+OR+(Colon,+Irritable[Title/Abstract]))+OR+(Irritable+Colon[Title/Abstract]))&sort=) | 05:21:51 |
| #2 |  |  | Search: ****((((((((Irritable Bowel Syndromes[Title/Abstract]) OR (Syndrome, Irritable Bowel[Title/Abstract])) OR (Syndromes, Irritable Bowel[Title/Abstract])) OR (Colitis, Mucous[Title/Abstract])) OR (Colitides, Mucous[Title/Abstract])) OR (Mucous Colitides[Title/Abstract])) OR (Mucous Colitis[Title/Abstract])) OR (Colon, Irritable[Title/Abstract])) OR (Irritable Colon[Title/Abstract])**** | [598](https://pubmed.ncbi.nlm.nih.gov/?term=((((((((Irritable+Bowel+Syndromes[Title/Abstract])+OR+(Syndrome,+Irritable+Bowel[Title/Abstract]))+OR+(Syndromes,+Irritable+Bowel[Title/Abstract]))+OR+(Colitis,+Mucous[Title/Abstract]))+OR+(Colitides,+Mucous[Title/Abstract]))+OR+(Mucous+Colitides[Title/Abstract]))+OR+(Mucous+Colitis[Title/Abstract]))+OR+(Colon,+Irritable[Title/Abstract]))+OR+(Irritable+Colon[Title/Abstract])&sort=) | 05:18:40 |
| #1 |  |  | Search: ****"Irritable Bowel Syndrome"[Mesh]**** Sort by: ****Most Recent**** |  |  |

**EMBASE:**

**#19**

#7 AND #17 AND #18

[268](http://www--embase--com--https.embase.shd1rmyy.lwnote.com:50001/)

**#18**

**'randomized controlled trial'**:ab,ti OR **'randomized'**:ab,ti OR **'placebo'**:ab,ti OR **'rct'**:ab,ti

[1,529,338](http://www--embase--com--https.embase.shd1rmyy.lwnote.com:50001/)

**#17**

#10 OR #13 OR #16

[2,151](http://www--embase--com--https.embase.shd1rmyy.lwnote.com:50001/)

**#16**

#14 OR #15

[455](http://www--embase--com--https.embase.shd1rmyy.lwnote.com:50001/)

**#15**

**'trulance'**:ab,ti OR **'sp-304'**:ab,ti

[18](http://www--embase--com--https.embase.shd1rmyy.lwnote.com:50001/)

**#14**

**plecanatide**

[452](http://www--embase--com--https.embase.shd1rmyy.lwnote.com:50001/)

**#13**

#11 OR #12

[1,658](http://www--embase--com--https.embase.shd1rmyy.lwnote.com:50001/)

**#12**

**'md-1100 acetate'**:ab,ti OR **'linaclotide acetate'**:ab,ti OR **'linzess'**:ab,ti OR **'md-1100'**:ab,ti OR **'asp-0456'**:ab,ti OR **'asp0456'**:ab,ti

[56](http://www--embase--com--https.embase.shd1rmyy.lwnote.com:50001/)

**#11**

**linaclotide**

[1,654](http://www--embase--com--https.embase.shd1rmyy.lwnote.com:50001/)

**#10**

#8 OR #9

[422](http://www--embase--com--https.embase.shd1rmyy.lwnote.com:50001/)

**#9**

**guanylate** AND **cyclase** AND **c** AND **agonists**

[364](http://www--embase--com--https.embase.shd1rmyy.lwnote.com:50001/)

**#8**

**guanylyl** AND **cyclase** AND **c** AND **agonists**

[153](http://www--embase--com--https.embase.shd1rmyy.lwnote.com:50001/)

**#7**

#3 AND #6

[7,637](http://www--embase--com--https.embase.shd1rmyy.lwnote.com:50001/)

**#6**

#4 OR #5

[142,393](http://www--embase--com--https.embase.shd1rmyy.lwnote.com:50001/)

**#5**

**'colonic inertia'**:ab,ti OR **'dyschezia'**:ab,ti

[1,337](http://www--embase--com--https.embase.shd1rmyy.lwnote.com:50001/)

**#4**

**constipation**

[142,057](http://www--embase--com--https.embase.shd1rmyy.lwnote.com:50001/)

**#3**

#1 OR #2

[32,210](http://www--embase--com--https.embase.shd1rmyy.lwnote.com:50001/)

**#2**

**'irritable bowel syndromes'**:ab,ti OR **'syndrome, irritable bowel'**:ab,ti OR **'syndromes, irritable bowel'**:ab,ti OR **'colitis, mucous'**:ab,ti OR **'colitides, mucous'**:ab,ti OR **'mucous colitides'**:ab,ti OR **'mucous colitis'**:ab,ti OR **'colon, irritable'**:ab,ti OR **'irritable colon'**:ab,ti

[783](http://www--embase--com--https.embase.shd1rmyy.lwnote.com:50001/)

**#1**

**irritable** AND (**'bowel'**/exp OR **bowel**) AND (**'syndrome'**/exp OR **syndrome**)

[31,735](http://www--embase--com--https.embase.shd1rmyy.lwnote.com:50001/)

**WOS：**

5

**#1 AND #2 AND #3 AND #4**

[189](https://www.webofscience.com/wos/woscc/summary/d897b07c-ddca-4161-ac47-c1aceb665d51-017820e3ec/relevance/1)

4

**TS=(randomized controlled trial OR randomized OR Placebo OR RCT)**

[1,367,620](https://www.webofscience.com/wos/woscc/summary/014632e6-f247-4594-b50a-ca6f52860db0-017820e2ca/relevance/1)

3

**TS=(Guanylyl Cyclase C Agonists OR Guanylate Cyclase C Agonists OR Linaclotide OR MD-1100 acetate OR Linaclotide Acetate OR Linzess OR MD-1100 OR ASP-0456 OR ASP0456 OR Plecanatide OR Trulance OR SP-304)**

[1,352](https://www.webofscience.com/wos/woscc/summary/e9a5c2b2-969f-4ccb-8730-3f3a9e71d060-017820e169/relevance/1)

2

**TS=(Constipation OR Colonic Inertia OR Dyschezia)**

[35,601](https://www.webofscience.com/wos/woscc/summary/5220ed6e-e5fa-418b-899b-8f59d4a69ad7-017820e011/relevance/1)

1

**TS=(Irritable Bowel Syndrome OR Irritable Bowel Syndromes OR Syndrome, Irritable Bowel OR Syndromes, Irritable Bowel OR Colitis, Mucous OR Colitides, Mucous OR Mucous Colitides OR Mucous Colitis OR Colon, Irritable OR Irritable Colon)**

[30,057](https://www.webofscience.com/wos/woscc/summary/c57ad68a-26f2-4c27-b4db-940e691a72ae-017820ddef/relevance/1)

**COCHRANE LIBRARY:**


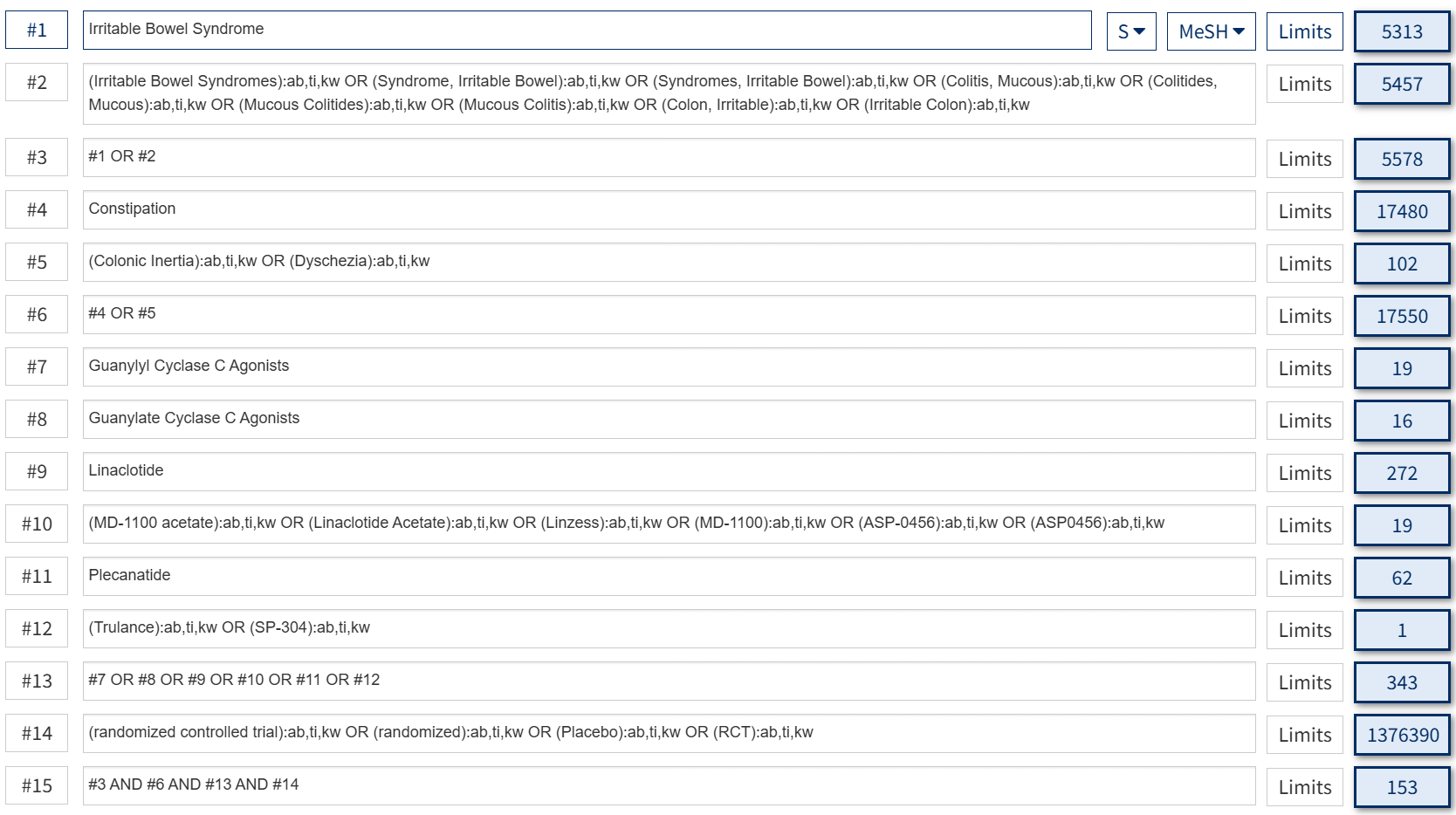


窗体底端
