# Supplementary material for: Efficacy and safety of Guanylyl cyclase C agonists (linaclotide and plecanatide) in patients with irritable bowel syndrome with constipation: a systematic review and meta-analysis of randomized controlled trials
Source: Front Pharmacol. 2026 Apr 10;17:1761301. doi: 10.3389/fphar.2026.1761301 (PMC13106150; doi:10.3389/fphar.2026.1761301)
Supplement: Supplementary file 4 [file Table3.docx]

**Author(s):** Zihao Zhou

**Question:** Guanylate cyclase-C (GCC) agonists compared to placebo for irritable bowel syndrome with constipation (IBS-C)

**Setting:**

**Bibliography: Cochrane Database of Systematic Reviews [Year], Issue [Issue].**

| **Certainty assessment** | | | | | | | **№ of patients** | | **Effect** | | **Certainty** | **Importance** |
| --- | --- | --- | --- | --- | --- | --- | --- | --- | --- | --- | --- | --- |
| **№ of studies** | **Study design** | **Risk of bias** | **Inconsistency** | **Indirectness** | **Imprecision** | **Other considerations** | **guanylate cyclase-C (GCC) agonists** | **placebo** | **Relative (95% CI)** | **Absolute (95% CI)** |  |  |
| **each week, ≥ 30 % decrease in worst abdominal pain + an increase ≥ 1 CSBM from baseline for at least 6 / 12 weeks** | | | | | | | | | | | | |
| 8 | randomised trials | not serious | not serious | not serious | not serious | none | 944/3137 (30.1%) | 433/2414 (17.9%) | **RR 1.71** (1.56 to 1.88) | **127 more per 1,000** (from 100 more to 158 more) | ⨁⨁⨁⨁ High | CRITICAL |
| **patients with ≥ 30 % decrease in abdominal pain for at least 6 / 12 weeks** | | | | | | | | | | | | |
| 6 | randomised trials | not serious | not serious | not serious | not serious | none | 1113/2634 (42.3%) | 600/1905 (31.5%) | **RR 1.39** (1.29 to 1.49) | **123 more per 1,000** (from 91 more to 154 more) | ⨁⨁⨁⨁ High | IMPORTANT |
| **patients w / CSBM rate increase ≥ 1 per week for at least 6 / 12 weeks** | | | | | | | | | | | | |
| 6 | randomised trials | not serious | not serious | not serious | not serious | none | 1183/2634 (44.9%) | 565/1905 (29.7%) | **RR 1.49** (1.32 to 1.68) | **145 more per 1,000** (from 95 more to 202 more) | ⨁⨁⨁⨁ High | IMPORTANT |
| **SBM ≤ 24h after first dose** | | | | | | | | | | | | |
| 5 | randomised trials | not serious | not serious | not serious | not serious | none | 1093/2263 (48.3%) | 492/1548 (31.8%) | **RR 1.52** (1.41 to 1.63) | **165 more per 1,000** (from 130 more to 200 more) | ⨁⨁⨁◯ Moderate^a^ | IMPORTANT |
| **change from baseline over the 12-week Treatment Period in abdominal pain (assessed with: 11-point NRS scale; Scale from: 0 to 10)** | | | | | | | | | | | | |
| 8 | randomised trials | not serious | not serious | not serious | not serious | none | 3137 | 2409 | - | MD **0.62 lower** (0.72 lower to 0.51 lower) | ⨁⨁⨁⨁ High | IMPORTANT |
| **change from baseline over the 12-week Treatment Period in CSBM Frequency Rate (CSBMs/Week)** | | | | | | | | | | | | |
| 9 | randomised trials | not serious | serious^a^ | not serious | not serious | none | 3219 | 2494 | - | MD **1.1 higher** (0.78 higher to 1.41 higher) | ⨁⨁⨁◯ Moderate^a^ | IMPORTANT |
| **patients with diarrhea over the 12-week Treatment Period** | | | | | | | | | | | | |
| 9 | randomised trials | not serious | not serious | not serious | not serious | none | 302/3219 (9.4%) | 43/2493 (1.7%) | **RR 5.54** (4.08 to 7.54) | **78 more per 1,000** (from 53 more to 113 more) | ⨁⨁⨁⨁ High | IMPORTANT |

**CI:** confidence interval; **MD:** mean difference; **RR:** risk ratio

#### Explanations

a. High heterogeneity
